# Cystic duct disimpaction for acute cholecystitis in the high-risk cholecystectomy patient: Case report

**DOI:** 10.1177/2050313X241232262

**Published:** 2024-02-14

**Authors:** Humzah Iqbal, Aalam Sohal, Kanana Aburayyan, Bandhul Hans, Juliana Yang

**Affiliations:** 1 Department of Internal Medicine, University of California San Francisco, Fresno, CA, USA; 2 Liver Institute Northwest, Seattle, WA, USA; 3 Department of Gastroenterology and Hepatology, University of Texas Medical Branch, Galveston, TX, USA

**Keywords:** Acute cholecystitis, cystic duct, choledocholithiasis, ERCP

## Abstract

Acute cholecystitis is a common cause of Emergency Department presentation and hospital admission. It is usually treated with early surgical removal of the gallbladder; however, some patients may not be fit to undergo the procedure due to critical illness or comorbidities. In these patients, options are limited. Endoscopic retrograde cholangiopancreatography interventions in this population are not well-studied. We present a case of a high-risk 59 year old female patient with a history of end-stage renal disease, heart failure, hypertension, pulmonary hypertension, and type 2 diabetes who presented with acute cholecystitis. She was successfully treated with cystic duct disimpaction without stenting, and continues to do well post-procedure with complete resolution of symptoms and abnormal lab findings.

## Introduction

Gallstone disease is a highly prevalent condition that affects approximately 10%–15% of the adult population.^1^ Most cases remain asymptomatic; however, up to 25% of patients develop symptoms.^2^ Symptomatic cholelithiasis may progress to acute cholecystitis (AC), which affects approximately 200,000 patients per year in the United States. AC is caused by cystic duct obstruction in 90%–95% of cases.^3^ If left untreated, AC can progress to abscess, peritonitis, septic shock, and death. Treatment of choice in most cases is laparoscopic cholecystectomy within 72 h.^4^ Young, healthy patients generally tolerate the procedure well; however, critically ill patients and those with serious comorbidities are poor surgical candidates and may warrant alternative treatments. Percutaneous cholecystostomy tube is often used as a bridge to surgery in high risk patients; however is not a definitive treatment for AC.^5^ Endoscopic retrograde cholangiopancreatography (ERCP) is a validated treatment modality for choledocholithiasis, with a short-term complication rate of 5%–10%.^6^ ERCP interventions for high-risk patients with AC due to cystic duct impactions are not well-studied. We describe a case of a high-risk cholecystectomy patient who underwent ERCP with cystic duct disimpaction, which led to resolution of cholecystitis.

## Case presentation

**Figure 1.**
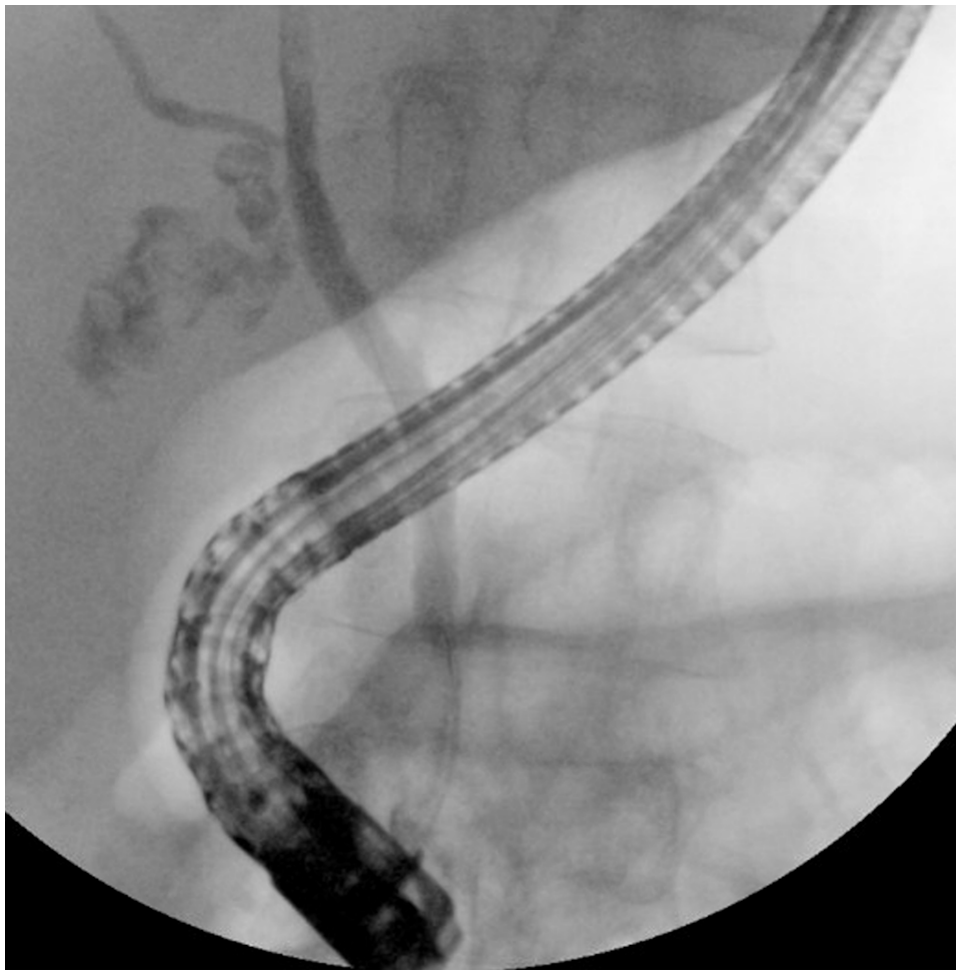
Endoscopic retrograde cholangiopancreatography demonstrating numerous gallstones and impacted cystic duct.

**Figure 2.**
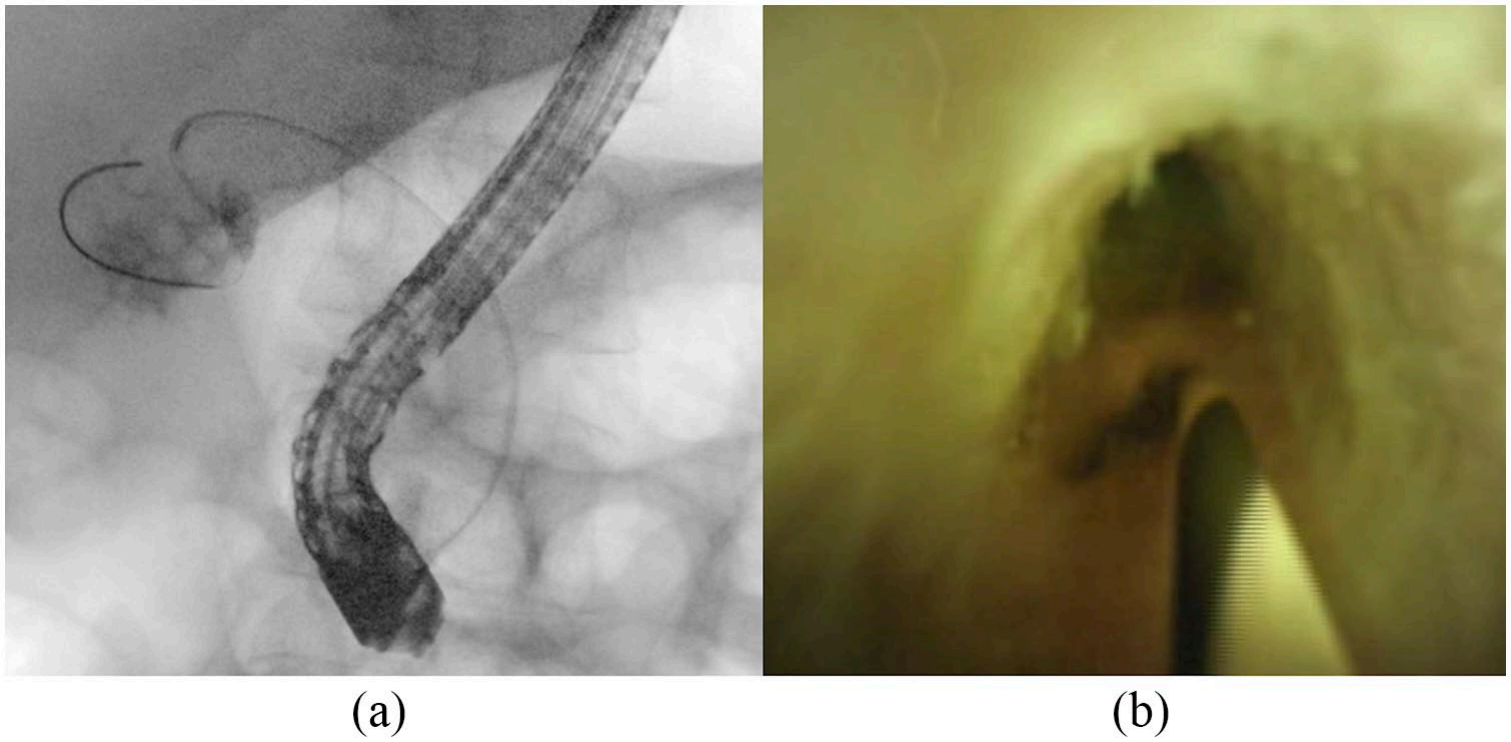
Cystic duct cannulation and stone removal, fluoroscopic (a) and endoscopic (b).

**Figure 3.**
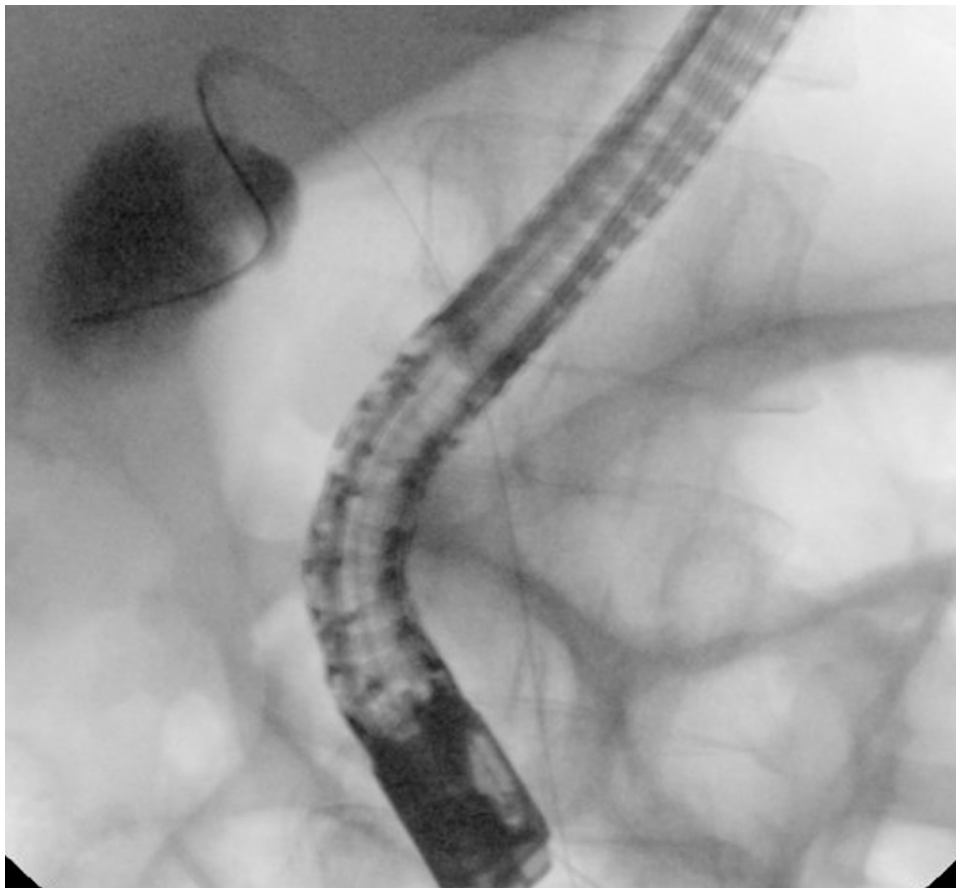
Post-cystic duct disimpaction.

A 59-year-old female patient with a history of end-stage renal disease on haemodialysis, heart failure, hypertension, pulmonary hypertension, and type 2 diabetes was admitted for worsening cough due to rhinovirus. On admission, she was noted to have nausea, vomiting, right upper quadrant pain and positive Murphy’s sign.^7^ She was afebrile with a white blood cell count of 5.09 10^3^/uL (normal: 4.3–11.1 10^3^/uL) and C-reactive protein of 12.7 mg/dL (normal: <0.8). Liver function showed acute elevation of total bilirubin 10.3 (0.1–1.1 mg/dL) from baseline of 1.2, aspartate transaminase 35 (13–40 U/L), alanine aminotransferase 33 (5–35 U/L), alkaline phosphatase 258 (34–122 U/L). Computed tomography showed distended gallbladder filled with gallstones and mild haziness of the gallbladder wall. Ultrasound showed gallbladder lumen filled with stones and pericholecystic fluid concerning for AC. Due to concern for choledocholithiasis, the patient underwent endoscopic ultrasound which revealed a 4.2 millimeter (mm) round stone in the cystic duct and numerous gallstones. ERCP was performed with stone removal, and numerous gallstones and cystic duct stones were visualized on fluoroscopy (Figure 1). Due to the patient’s numerous comorbidities, she was deemed high-risk for surgical cholecystectomy despite having mild cholecystitis. The patient was offered but refused percutaneous gallbladder drainage. Due to ongoing symptomatic cholecystitis despite antibiotic use, a repeat ERCP was performed under general anaesthesia with the goal to disimpact the cystic duct with transcapillary gallbladder drainage using cholangioscopy. The cystic duct was cannulated under direct cholangioscopic visualization. The gallbladder and the cystic duct were opacified and multiple filling defects were visualized. Using a combination of water flushes and sphincterotome manipulations, multiple cystic duct stones were removed. A transpapillary gallbladder stent was attempted but not placed due to acute cystic duct angulation and tortuous anatomy (Figure 2). Repeat cholangiogram showed no retained stones in the cystic duct (Figure 3). The patient’s abdominal pain resolved post-procedure and she did not require percutaneous gallbladder drain placement. The patient was subsequently discharged with 10 days of antibiotics. She continues to do well 1 month post procedure.

## Discussion

Surgical cholecystectomy is the gold standard of treatment for AC; however, management of AC in high-risk patients can be challenging. Laparoscopic cholecystectomy in high-risk candidates has been associated with a serious morbidity rate of 41% and mortality rate of 4.5%.^8,9^ Percutaneous cholecystostomy is sometimes used in high-risk individuals; however, the CHOCOLATE trial (Dutch Trial Register NTR2666) demonstrated that early cholecystectomy is associated with a lower rate of serious complications and lower resource utilization compared to percutaneous cholecystostomy among high-risk patients. Additionally, percutaneous drainage is not definitive, and can lead to recurrent cholecystitis, readmissions, and increased mortality.^5^ Our patient was offered percutaneous drainage as the next step in management, which was however declined since she did not want an external drain. Given the mildness of her cholecystitis and stability for ERCP as she was not septic or decompensating, the decision was made to perform ERCP for definitive management and potentially avoid cholecystectomy.

Data is limited regarding ERCP interventions for high-risk cholecystectomy patients. A case report by Bonner et al. describes cystic duct disimpaction with cholecystoduodenal stenting for a high-risk patient with AC.^10^ Brown et al. also described resolution of cholecystitis with metallic stent placement into the cystic duct in five patients.^11^ Stent placement into the cystic duct is a technically challenging procedure. Presence of ductal strictures may prevent guidewire access. Stents may become occluded leading to recurrent biliary obstruction or may impede future biliary access. Additionally, biliary stents can migrate and cause perforation, leading to further complications.^10^ A study of 33 patients by Hersey et al. found a 91% technical success rate and 10% 30-day complication rate among patients undergoing cystic duct stenting for cholecystitis.^12^ In our patient, stenting was deferred due to patient’s anatomy. This may help to avoid potential complications and the need for repeat procedures in case of stent migration or occlusion. Resolution of cholecystitis in our case was achieved with disimpaction of the cystic duct, which may represent a viable treatment option in cases where surgical management is not feasible. Stent placement is an option; however, it may not be necessary or achievable in every scenario.

## Conclusion

AC is a common presentation and frequent cause of hospital admission that can lead to sepsis and death if left untreated. Though surgical cholecystectomy is the most common and well-studied treatment option, not all patients are suitable to undergo the procedure. In such cases, ERCP interventions for AC such as cystic duct disimpaction should be considered. We present such a case in a 59 year old female with significant cardiovascular and renal comorbidities. Surgical management was deferred, external drainage was declined, and she experienced complete resolution of her cholecystitis following ERCP intervention. Further studies are needed in order to assess the technical and clinical success of ERCP interventions for high-risk surgical candidates with AC.
